# *Fusobacterium nucleatum*-triggered purine metabolic reprogramming drives tumorigenesis in head and neck carcinoma

**DOI:** 10.1007/s12672-023-00727-x

**Published:** 2023-07-02

**Authors:** Feiran Li, Huiying Huang, Jing Xu, Lei Tao, Liang Zhou, Chiyao Hsueh, Hongli Gong, Ming Zhang

**Affiliations:** 1 Department of Otorhinolaryngology, Eye & ENT Hospital, Fudan University, 83 Fen Yang Road, Shanghai, China; 2 Department of Nursing, Eye & ENT Hospital, Fudan University, 83 Fen Yang Road, Shanghai, China

**Keywords:** Head and neck carcinoma, *Fusobacterium nucleatum*, Metabolic reprogramming, Purine metabolic pathway, Prognosis

## Abstract

**Background:**

*Fusobacterium nucleatum* (*F. nucleatum*) is a vital pro-oncogenic bacterium. Our previous study revealed that a high abundance of *F. nucleatum* in head and neck squamous cell carcinoma (HNSCC) is correlated with poor patient prognosis. However, the impact of *F. nucleatum* on metabolic reprogramming and tumor progression in HNSCC awaits more exploration.

**Methods:**

Liquid chromatography‒mass spectrometry (LC‒MS) was applied to analyze the altered metabolites in a head and neck carcinoma cell line (AMC-HN-8) after coculture with *F. nucleatum* for 24 hrs and 48 hrs. Both univariate and multivariate analyses were used to screen for differential metabolites. Kyoto Encyclopedia of Genes and Genomes (KEGG) metabolic pathway enrichment analysis was further used to explore the metabolic changes.

**Results:**

We observed a significantly altered metabolic profile in AMC-HN-8 cells over time after coculture with *F. nucleatum*. Among the several enriched pathways, the purine metabolic pathway was the most significantly enriched (*P* = 0.0005), with downregulation of purine degradation. Furthermore, uric acid, the end product of purine metabolism, significantly reversed *F. nucleatum*-triggered tumor progression and altered the intracellular reactive oxygen species (ROS) level. Moreover, the negative correlation between the serum uric acid level and the abundance of *F. nucleatum* was verified in 113 HNSCC patients (*P* = 0.0412, R = − 0.1924).

**Conclusions:**

Our study revealed obviously aberrant purine metabolism driven by *F. nucleatum* in HNSCC, which was closely related to tumor progression and patient prognosis. These findings indicate the possibility of targeting *F. nucleatum*-induced purine metabolism reprogramming in the future treatment of HNSCC.

## Introduction

Head and neck squamous cell carcinoma (HNSCC) is the seventh most common cancer worldwide, with more than 500,000 newly diagnosed cases and 400,000 related deaths annually [1]. Moreover, its incidence shows an increasing trend and is estimated to increase 30% by 2030 [2]. In the past several years, advances have been made in the therapeutic approaches for HNSCC, including surgery, radiotherapy and systemic therapy [3]. However, these promising treatments, either alone or in combination, have not significantly improved patient prognosis, and the five-year survival rate of HNSCC has remained approximately 50% [4]. Hence, it is still vital to reveal the molecular mechanisms involved in disease progression and to provide more evidence for identifying new therapeutic targets.

In recent years, the involvement of the microbiota in cancer progression has gained increasing attention. The head and neck is a part of the upper respiratory airway, which is a natural shelter for various microbial floras [5]. Among these microbes, *Fusobacterium* have a higher abundance in HNSCC tissues than in noncancerous tissues, as verified in our previous studies [6–9]. *Fusobacterium nucleatum* (*F. nucleatum*), an important species of *Fusobacterium*, is considered a pro-oncogenic bacterium that mediates the initiation, progression and chemoresistance of several alimentary and respiratory cancers [10, 11]. However, the mechanism underlying these findings in HNSCC awaits more exploration.

We observed altered ethanol metabolism in HNSCC induced by *F. nucleatum* accumulation and validated the negative effect of this feed-forward loop on patient survival [12]. As *F. nucleatum*-induced metabolic reprogramming plays an important role in tumor progression, we hypothesized that *F. nucleatum* may alter metabolic profiles in addition to ethanol metabolism in HNSCC. Thus, in this study, we further elucidate *F. nucleatum*-induced metabolic reprogramming of HNSCC and explore the potential impact of this metabolic crosstalk on disease development and patient prognosis.

## Method

### Tissue sample collection and *F. nucleatum* detection

The study was approved by the ethics committee of the Eye & ENT Hospital, Fudan University. A total of 113 tissue samples were obtained from HNSCC patients undergoing surgery at the Eye & ENT Hospital, Fudan University. Patients were pathologically diagnosed with HNSCC, the classification was confirmed according to the American Joint Committee on Cancer (eighth edition) cancer staging manual. The exclusion criteria were as follows: (i) distant metastasis, (ii) a concurrent second primary tumor, (iii) active infection, (iv) antibiotic use within the three months before surgery, and (v) preoperative treatment, including chemotherapy or radiotherapy. The patients’ clinical data are shown in Table 1.

**Table 1:** Patient clinical characteristics

	Number (%)
Total	113 (100)
Age	
> 65 years	44 (38.9)
≤ 65 years	69 (61.1)
Sex	
Male	111 (98.2)
Female	2 (1.8)
Smoking	
Yes	102 (90.3)
No	11 (9.7)
Drinking	
Yes	67 (59.3)
No	46 (40.7)
Subregion	
Supraglottis	44 (38.9)
Glottis	65 (57.5)
Subglottis	4 (3.5)
T classification	
T1/2	44 (38.9)
T3/4	69 (61.1)
N classification	
N0	75 (66.4)
N1	11 (9.7)
N2/3	27 (23.9)
C stage	
C1/2	34 (30.1)
C3/4	79 (69.9)

By using the primer set for the gene prostaglandin transporter (PGT) extracted from fresh tissues and paraffin embedded tissues as the reference, the cycle threshold (Ct) values of *F. nucleatum* DNA were normalized to the amount of human genomic DNA (gDNA) as described previously (Castellarin et al. [13]). *F. nucleatum* DNA was amplified and detected in a 96-well optical PCR plate with an ABI 7500 Real-Time PCR System (Thermo Fisher, Massachusetts, USA) as previously described (Hsueh et al. [9]). Then, the -Δ Ct method was applied for relative quantification.

### Cell and bacterial culture

Head and neck carcinoma cell lines, including AMC-HN-8 and FaDu, were generously given by Stem Cell Bank, Chinese Academy of Sciences and were cultured in RPMI-1640 medium (HyClone, Utah, USA) supplemented with 1% (v/v) penicillin‒streptomycin and 10% fetal bovine serum (FBS) under standard cell culture conditions (37 °C, 5% CO2). The *F. nucleatum* strain was purchased from the American Type Culture Collection (ATCC 25586, Virginia, USA) and was maintained overnight at 37 °C under anaerobic conditions on Columbia blood agar supplemented with five mg/mL hemin, 5% defibrinated sheep blood, and one mg/mL vitamin K1. AMC-HN-8 cells and *F. nucleatum* were then cocultured for 24 hs (Fn_24hr group) and 48 hs (Fn_48hr group) at a cell: bacteria ratio of 1:500 with six biological duplicates.

### Cell collection and extraction

Cells were washed with ice-cold PBS. After trypsin digestion and centrifugation, detached cells were collected and then immediately stored at − 80 °C until analysis. Stored samples were thawed slowly on ice. To extract intracellular metabolites, 500 µL of pure methanol (Merck, Germany) was added to the sample. The mixture was vortexed for 5 min (Jingxin MIX-200, Shanghai, China), and subjected to three freeze‒thaw cycles. After centrifugation at 4 °C for 10 min (Eppendorf 5427R, Germany), 300 µL of methanol containing the internal standard was added to the sample. The mixture was then vortexed for one min and placed in a − 20 °C freezer for one hr. Then, the sample was centrifuged at 12,000 rpm at 4 °C for 10 min. 200 µL of the supernatant was retained and allowed to stand for 30 min. Then, centrifugation was repeated under the same conditions. The collected supernatant was then stored in a sample injection bottle for further analysis.

### Liquid chromatography‒mass spectrometry (LC‒MS) analysis

Chromatographic analysis was performed using an ultra-high-performance liquid chromatography (UHPLC) system (Agilent 1290 INFINITY, USA). The mobile phases consisted of water with 0.04% acetic acid (solution A) (Thermo Fisher, USA) and acetonitrile (Merck, Germany) with 0.04% acetic acid (solution B). The column temperature was maintained at 40 °C. The flow rate was 0.35 mL/min, and the sample injection volume was 2 µL. Analysis was performed in both negative and positive ion modes with a Q-TOF mass spectrometer (Agilent 6545, USA). The voltages were 250 and 1500 in ESI − and ESI ^+^ modes, respectively. The other parameters were the same in bodes modes, and the settings were as follows: gas flow rate, 8; fragmentor voltage, 135; gas temperature, 325; sheath temperature, 325; sheath flow rate, 11; nebulizer gas pressure, 40. To examine the repeatability of the samples with the same treatment, we prepared samples for quality control by mixing equal amounts of extracts from the experimental samples. To further monitor the analysis’ stability, A quality control sample was inserted into every ten samples throughout the whole analysis.

### Data extraction and compound identification

The original mass spectrometry data were converted into mzML format with ProteoWizard. The XCMS program was used for peak identification, peak alignment and retention time correction. The peak area was corrected by the SVR method. For every sample group, the peaks were filtered based on a deletion rate of greater than 50%. Then, metabolic information was identified by searching the MetWare database (MetWare Biotechnology, Wuhan, China) and a public metabolite information database combined with the MetDNA algorithm.

### Cell proliferation, invasion, migration and intracellular ROS assays

The malignance of cancer cells is commonly evaluated by assessing their proliferation, migration and invasion. HNSCC cells were harvested at logarithmic growth phase and seeded into a 96-well plate at 3000 cells per well. Then, 10 μL Cell Counting Kit-8 (CCK8, Dojindo, Kumamoto, Japan) reagent was added to the culture medium and incubated with the sample for two hs. The optical density value at 450 nm were measured using a microplate reader (BioTek Instruments Inc., Vermont, USA). For the migration assay, 1 × 10^6^ HSCC cells were first cultured with or without *F. nucleatum* or 0.7 g/L uric acid for 72 hrs. Then, they were resuspended in serum-free medium and seeded into the upper chamber of Transwell inserts (Corning, New York, USA) containing a membrane with an eight µm pore size. The bottom chamber contained RPMI-1640 medium or BEGM supplemented with 10% FBS. After an additional 24 hrs of incubation, the inserts were removed. The cells were washed with PBS, fixed with 4% methanol, and stained using crystal violet. Three fields/insert were randomly selected under a light microscope to count the stained cells. The cell invasion assay was performed using Transwell chambers containing Matrigel-coated membranes (BD Biosciences). Intracellular ROS levels were measured using a fluorometric intracellular ROS assay kit (Sigma). One microliter of ROS detection reagent stock solution was added to the cell medium per mL of cells, and the cells were incubated in a 5% CO2 incubator at 37 °C for 30 min. Then, images were acquired using fluorescence microscopy, and fluorescence intensity values were determined using ImageJ software (National Institutes of Health, USA).

### Statistical analysis

To identify differential metabolites between the groups, both univariate and multivariate analyses were performed. The univariate analyses included Student’s t test and fold change analysis, and the multivariate analyses included principal component analysis (PCA) and orthogonal partial least squares discriminant analysis (OPLS-DA). PCA is an unsupervised multidimensional statistical analysis that was applied to visualize the overall metabolic differences and the variability within the groups. The quality control samples are labeled as “mix” in the PCA plot. OPLS-DA is a supervised multivariate analysis that maximizes the variance between groups. The variable importance in projection (VIP) value was calculated to summarize the relative importance of each X variable to the association between the X and Y variables. A VIP value of ≥ 1 indicated that the metabolite had a statistically significant contribution to the model. For correlation analysis, Pearson or Spearman correlation analysis was applied depending on the normality of the data. *P* < 0.05 was considered statistically significant.

## Results

### *F. nucleatum* significantly alters purine metabolism in AMC-HN-8 cells

**Fig. 1:**
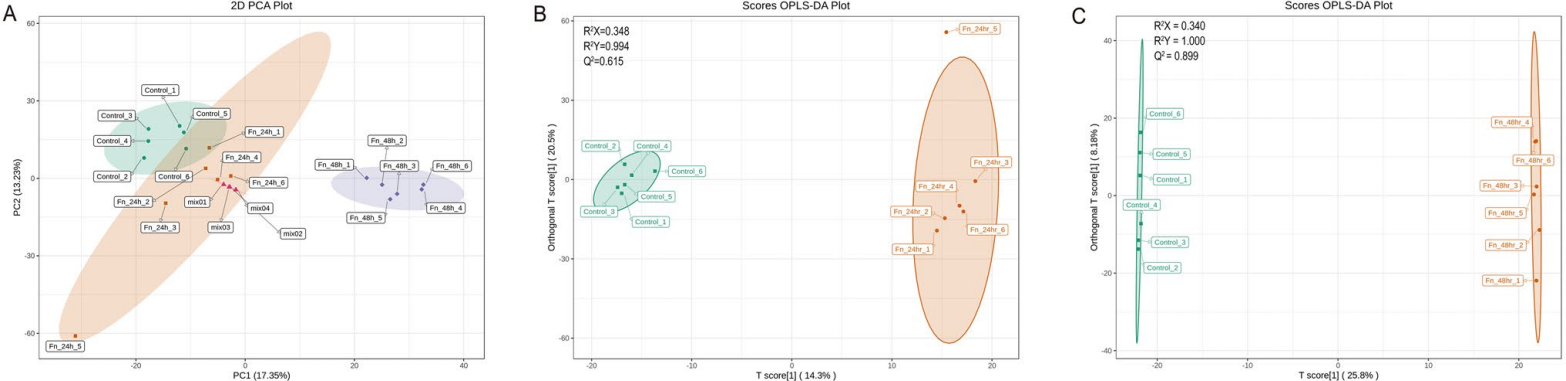
Overall metabolic profiles of control, Fn_24hr group and Fn_48hr groups. **A** principal component analysis (PCA) plot for three groups was performed to visualize the overall metabolic differences and the variability within the groups. **B** orthogonal partial least squares discriminant analysis (OPLS-DA) plot of Fn_24hr group and control group, and **C** OPLS-DA of Fn_48hr group and control group were performed to further track the metabolic changes, which maximizes the variance between groups. Fn_24hr group: AMC-HN-8 cells and *F. nucleatum* were cocultured for 24hs. Fn_48hr group: AMC-HN-8 cells and *F. nucleatum* were cocultured for 48hs

We explored changes in the overall metabolic profile of AMC-HN-8 cells after coculture with *F. nucleatum*. The PCA results showed evident separation among the three groups without outliers detected in the overview (Fig. 1A). Additionally, there were no obvious differences among the quality control samples, indicating that the system was stable for analysis with good repeatability. OPLS-DA was then performed to further track the metabolic changes in AMC-HN-8 cells after coculture with *F. nucleatum*. The OPLS-DA model achieved a good overall model fit (R^2^Y = 0.994 and 1.000 for the Fn_24hr group and Fn_48hr group, respectively) and prediction ability (Q^2^ = 0.615 and 0.899 for the Fn_24hr group and Fn_48hr group, respectively) (Fig. 1B, C). Therefore, in the initial assessment, we observed obviously different metabolic profiles in the Fn_48hr group, Fn_24hr group and control group, indicating that *F. nucleatum* can interfere with metabolism in AMC-HN-8 cells.

**Fig. 2:**
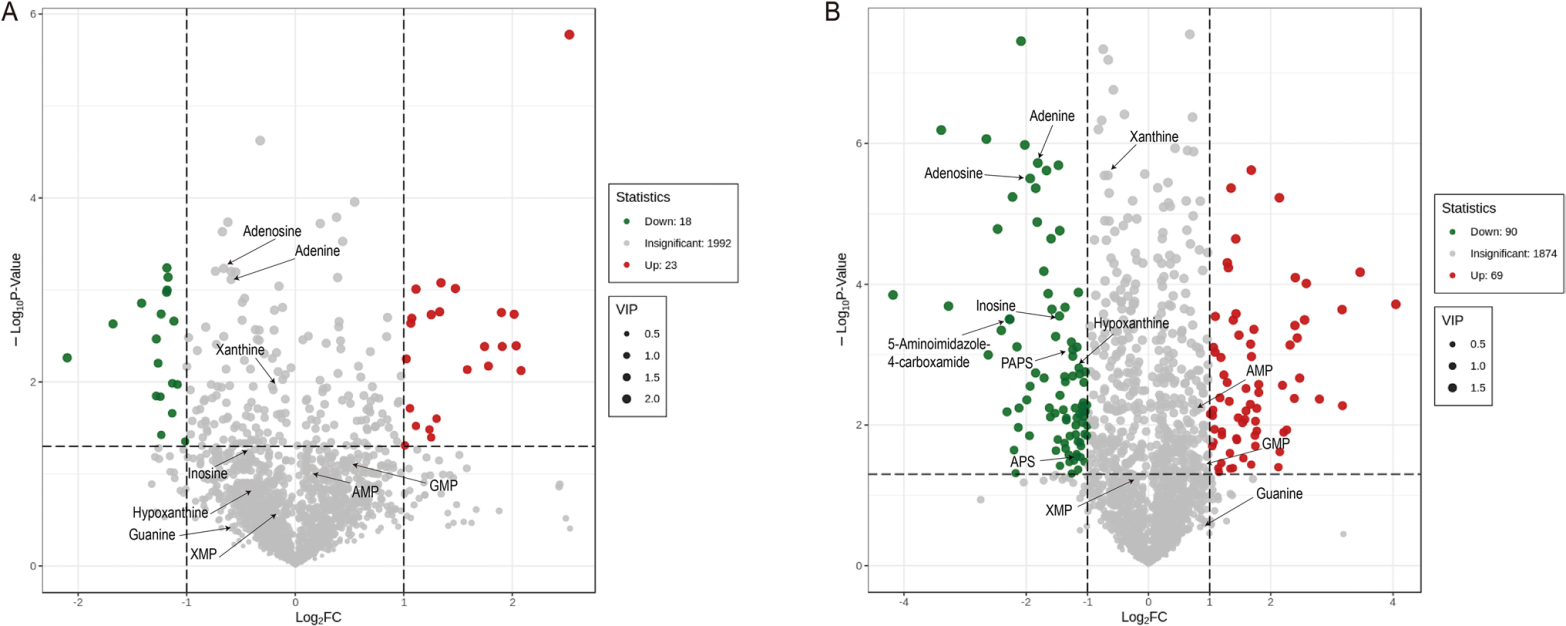
Volcano plot showing metabolic alterations of (**A**) Fn_24hr group vs. control group and (**B**) Fn_48hr group vs. control group. Arrows point out critical metabolites related to the purine metabolic pathway. Fn_24hr group: AMC-HN-8 cells and *F. nucleatum* were cocultured for 24 hs. Fn_48hr group: AMC-HN-8 cells and *F. nucleatum* were cocultured for 48 hs

After data extraction and processing, we detected and identified a total of 2033 metabolites in all samples. Significant metabolites were confirmed by combining the results of both univariate and multivariate analyses. Only metabolites with *P* < 0.05 on the t test, a fold change (FC) of ≥ 2 or ≤ 0.5 and a VIP of ≥ 1 were considered significantly altered. According to these criteria, 41 metabolites in the Fn_24hr group, namely, 23 upregulated and 18 downregulated metabolites, were identified in the comparison with the control group (Fig. 2A). More significantly altered metabolites, including 69 upregulated and 90 downregulated metabolites, were observed in the comparison of the Fn_48hr and control groups (Fig. 2B). Thus, we verified the significantly altered metabolites in AMC-HN-8 cells after coculture with *F. nucleatum*, as directly shown in the volcano plots.

**Fig. 3:**
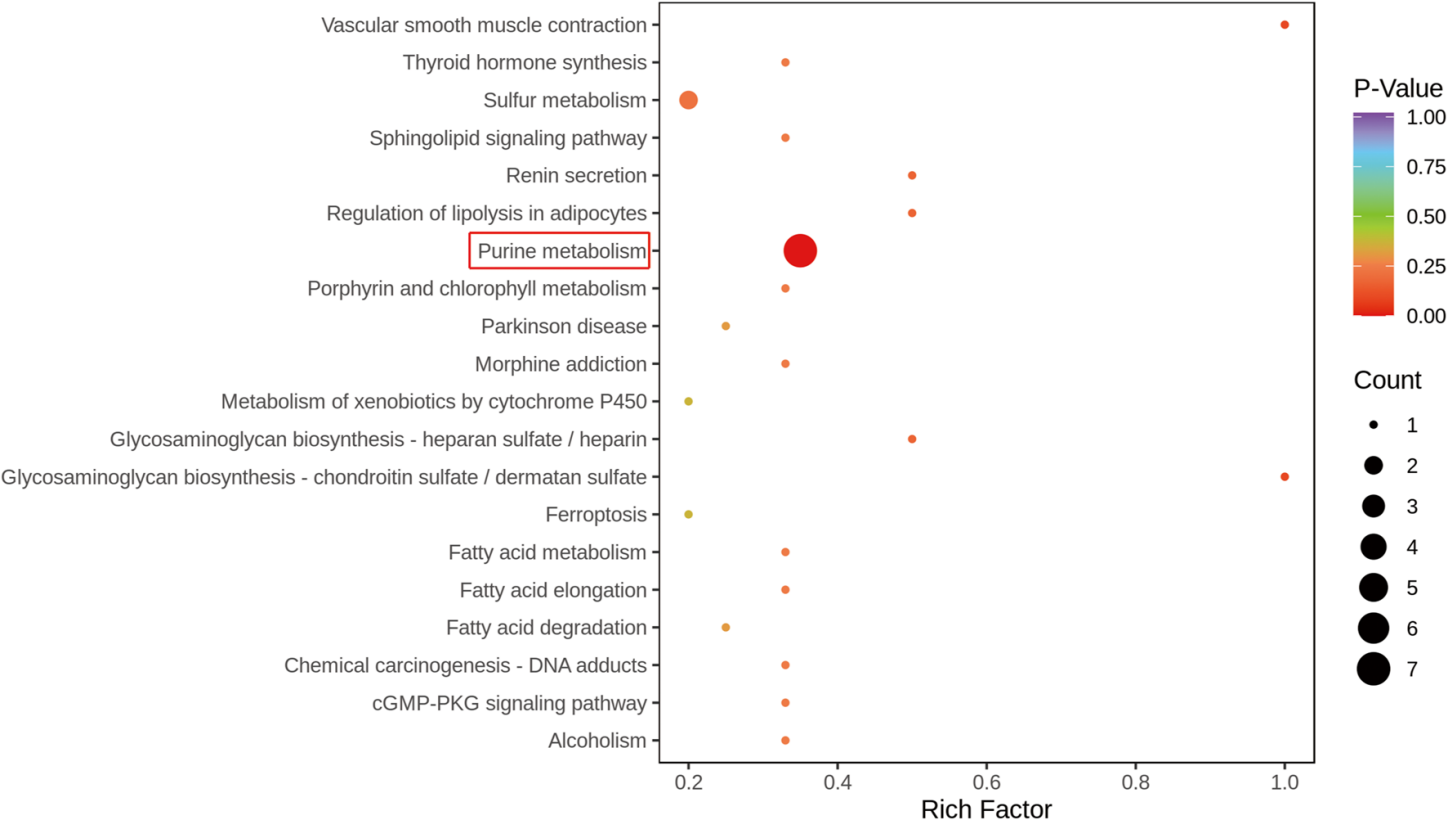
Metabolic pathway enrichment analysis for significantly altered metabolites for Fn_48hr group and control group. Purine metabolism was the most significant metabolic pathway, which enriched seven significantly altered metabolites

Candidate metabolites were then imported into the Kyoto Encyclopedia of Genes and Genomes (KEGG) analysis tool for metabolic pathway enrichment analysis. A total of 33 metabolic pathways were affected in AMC-HN-8 cells by *F. nucleatum* after 48 hs of coculture compared with control cells (Fig. 3). The top five metabolic pathways were purine metabolism, glycosaminoglycan biosynthesis–chondroitin sulfate/dermatan sulfate, vascular smooth muscle contraction, renin secretion and regulation of lipolysis in adipocytes (Table 2). The most significant metabolic pathway (*P* = 0.0005), purine metabolism, was enriched with 20 identifiable metabolites and seven significantly altered metabolites, namely, adenine, adenosine, hypoxanthine, inosine, phosphoadenosine phosphosulfate (PAPS), adenosine-5’-phosphosulfate (APS) and 5-aminoimidazole-4-carboxamide. These results indicate that purine metabolic reprogramming plays a dominant role in the metabolic crosstalk of *F. nucleatum* with AMC-HN-8 cells.

**Table 2:** Metabolic pathway enrichment distinguished by Fn_48hr and control

KEGG pathway	Cluster frequency		*P* value
Total	Significant metabolites	All metabolites	
	19 (100.00%)	217 (100.00%)	
Purine metabolism	7 (36.84%)	20 (9.22%)	0.0005
Glycosaminoglycan biosynthesis—chondroitin sulfate/dermatan sulfate	1 (5.26%)	1 (0.46%)	0.0876
Vascular smooth muscle contraction	1 (5.26%)	1 (0.46%)	0.0876
Renin secretion	1 (5.26%)	2 (0.92%)	0.1678
Regulation of lipolysis in adipocytes	1 (5.26%)	2 (0.92%)	0.1678

^*^: The table showed the top five metabolic pathways

### *F. nucleatum* causes downregulation of purine degradation in AMC-HN-8 cells

**Fig. 4:**
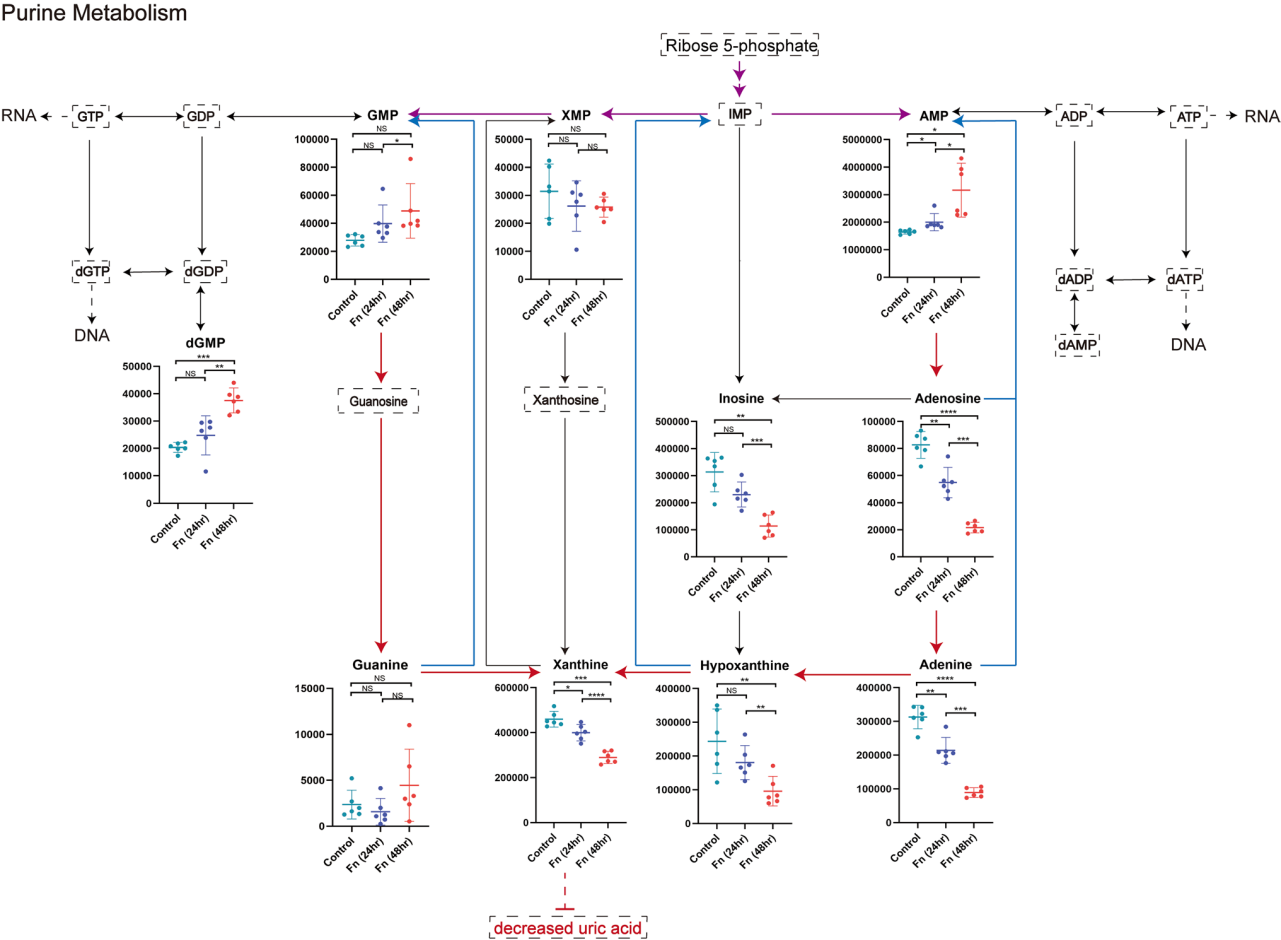
Overview of changed metabolites in purine metabolic pathway. Purple arrow: de novo biosynthetic pathway. Blue arrow: complementary salvage pathway. Red arrow: purine degradation pathway. Metabolites surrounded by dashed line were undetectable ones. *: *P* < 0.05. **: *P* < 0.01. ***: *P* < 0.001. ****: *P* < 0.0001

To further explore the altered purine metabolic pathways, we summarized all detected purine-related metabolites in Table 3 and showed the relationships among critical metabolites in Fig. 4. We found that compared to those in the control group, the levels of adenosine 5’-monophosphate (AMP) and guanosine 5’-phosphate (GMP), which are not only two key end-products of purine synthesis but also substrates for further synthesis of DNA and RNA, were increased over time (*P* = 0.0338 and 0.0101 for AMP, *P* = 0.1010 and 0.0274 for GMP, respectively). In addition to upregulation of purine synthesis, the downregulation of purine degradation is also observed in our study. Although uric acid could not be detected, the levels of its upstream metabolites involved in purine degradation, including xanthine (*P* = 0.0181 and 0.0002), hypoxanthine (*P* = 0.1597 and 0.0089) and adenine (*P* = 0.0067 and < 0.0001), were significantly decreased in a time-dependent manner compared to those in the control group. This result reveals that *F. nucleatum* can cause significant decreases in the levels of metabolites in the purine degradation pathway, which may provide essential purine nucleotides to support cancer cell proliferation.

**Table 3:** Difference analysis of metabolites related to purine metabolism pathway

Metabolites	Fn_24hr vs. control		Fn_48hr vs. control		Fn_48hr vs. Fn_24hr	
	*P* value*	FC	*P* value	FC	*P* value	FC
Adenine	0.0067	0.68	< 0.0001	0.28	0.0001	0.42
Adenosine	0.0095	0.66	< 0.0001	0.26	0.0001	0.39
Hypoxanthine	0.1597	0.74	0.0089	0.39	0.0022	0.53
Inosine	0.0627	0.74	0.0028	0.36	0.0002	0.50
PAPS	0.0485	0.65	0.0040	0.44	0.0626	0.68
APS	0.6943	0.92	0.0312	0.41	0.0092	0.44
AMP	0.0338	1.22	0.0101	1.92	0.0278	1.58
AppppA	0.0413	1.50	0.0091	1.95	0.2587	1.30
Sulfate	0.1383	1.21	0.9233	1.02	0.0542	0.84
Xanthine	0.0181	0.87	0.0002	0.63	< 0.0001	0.72
XMP	0.4004	0.83	0.2067	0.82	0.9208	0.99
Guanine	0.3620	0.67	0.0929	1.89	0.1056	2.82
GMP	0.1010	1.43	0.0274	1.75	0.4194	1.23
dGMP	0.2015	1.22	0.0002	1.85	0.0017	1.52
Allantoic acid	0.0633	1.07	0.5780	0.98	0.0112	0.91
5-Aminoimidazole-4-carboxamide	0.1549	0.70	0.0004	0.21	0.0039	0.30
ppGpp	0.2117	0.71	0.3996	1.14	0.0605	1.60
ADP-ribose	0.1783	0.90	0.0686	1.29	0.0149	1.43
5-Amino-4-imidazole carboxylate	0.1812	0.87	< 0.0001	0.57	0.0116	0.65
IDP	0.4088	1.31	0.2108	1.34	0.8791	1.03

^*^: *P* value was obtained from t-test

### *F. nucleatum*-triggered tumor progression and ROS accumulation can be reversed by uric acid

**Fig. 5:**
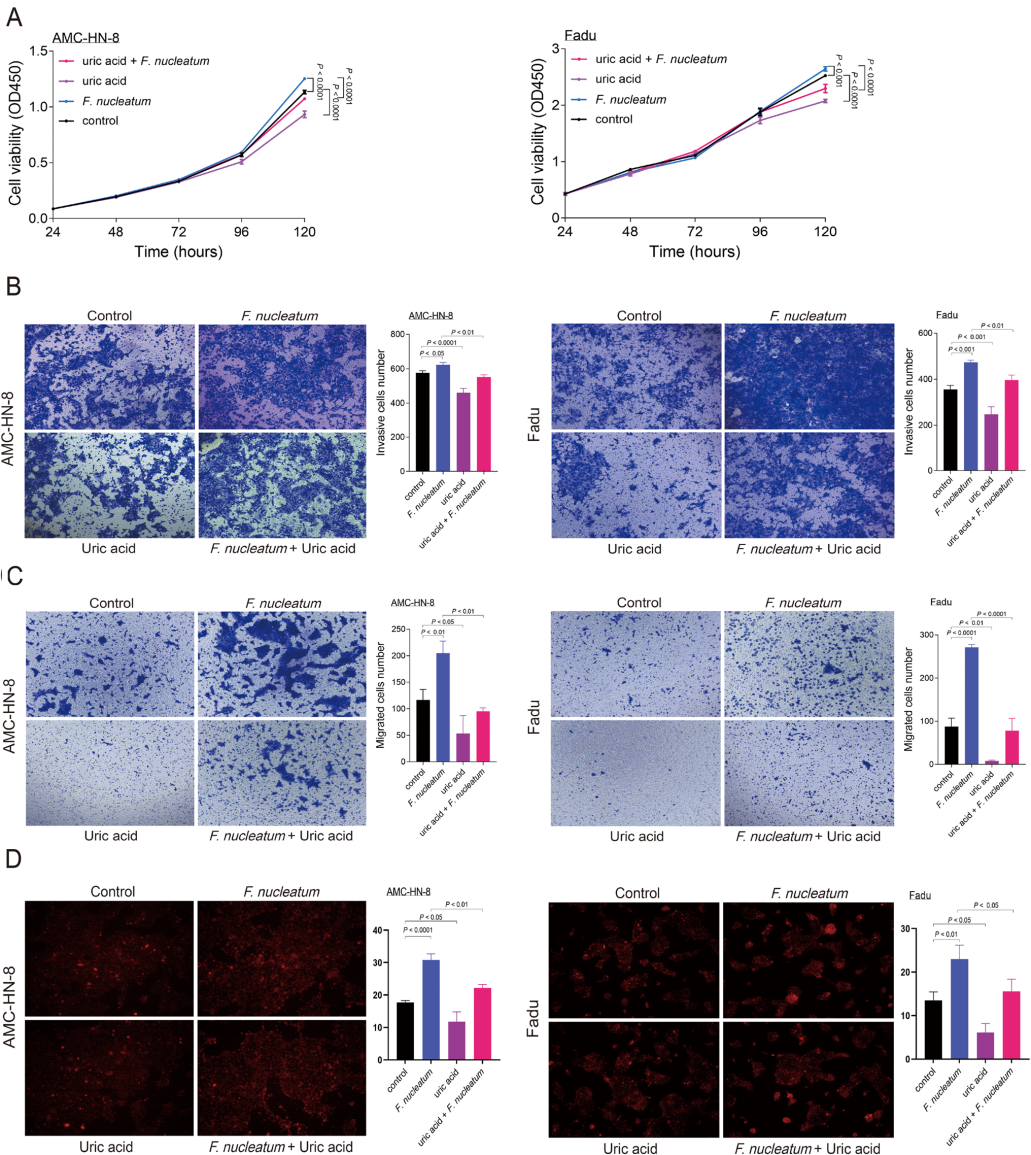
*F. nucleatum*–triggered tumor progression and ROS accumulation could be reversed by uric acid: **A** cell proliferation measured using Cell Counting Kit-8 assay (CCK8), **B** invasion assay performed using transwell chambers, **C** migration assay performed using transwell chambers and **D** Intracellular ROS detection using fluorometric intracellular ROS assay kit. Left: AMC-HN-8 cells. Right: Fadu cells

We then explored the roles of *F. nucleatum* and uric acid in HNSCC progression. Our results showed that *F. nucleatum* significantly promoted the proliferation (*P* < 0.0001 and *P* < 0.001 in AMC-HN-8 and FaDu cells, respectively) and facilitated the invasion (*P* = 0.0262, *P* = 0.0005) and migration (*P* = 0.0043, *P* < 0.0001) of both AMC-HN-8 and FaDu cells. In contrast, uric acid significantly inhibited tumor cell proliferation (*P* < 0.0001 and *P* < 0.0001 in AMC-HN-8 and FaDu cells, respectively), invasion (*P* < 0.0001, *P* = 0.0009) and migration (*P* = 0.0274, *P* = 0.0017). *F. nucleatum*-triggered tumor progression was reversed by uric acid, as indicated by the decreases in proliferation (*P* < 0.0001 and *P* < 0.0001 in AMC-HN-8 and FaDu cells, respectively), invasion (*P* = 0.0024, *P* = 0.0076) and migration (*P* = 0.0011, *P* < 0.0001) (Fig. 5A, B, C). Furthermore, *F. nucleatum* led to ROS accumulation (*P* < 0.0001 and *P* = 0.0056 in AMC-HN-8 and FaDu cells, respectively), whereas uric acid decreased intracellular ROS levels (*P* = 0.0161, *P* = 0.0217). *F. nucleatum*-triggered ROS accumulation was reversed by uric acid (*P* = 0.0016, *P* = 0.0212) (Fig. 5D). Thus, uric acid may reverse *F. nucleatum*-triggered tumor progression by decreasing intracellular ROS levels.

### *F. nucleatum* is negatively correlated with the serum uric acid level and leads to poor prognosis in patients

**Fig. 6:**
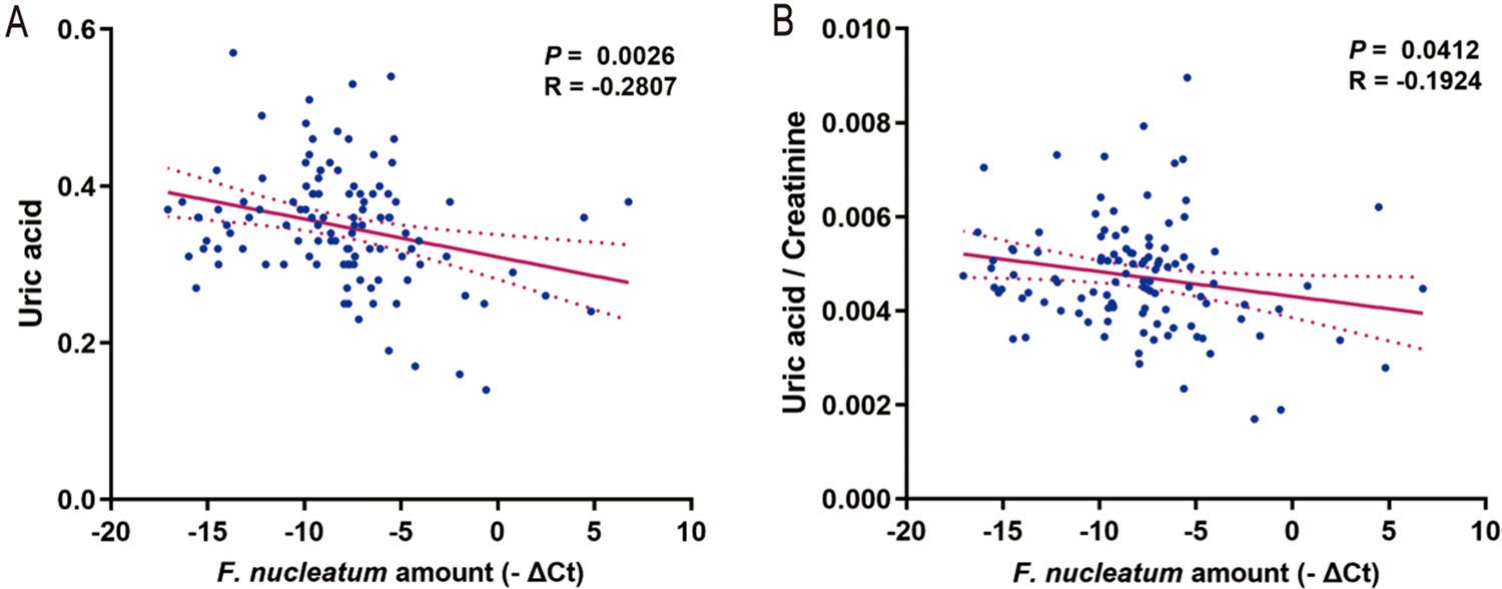
Correlation analysis of *F. nucleatum* abundance and uric acid level in head and neck squamous cell carcinoma (HNSCC) patients. **A** *F. nucleatum* and uric acid correlation analysis, **B** *F. nucleatum* and uric acid/creatinine correlation analysis

We then explored the correlation between the serum uric acid level and *F. nucleatum* abundance in 113 patients diagnosed with HNSCC. Patients’ serum was collected within one month before surgery. The results showed that the uric acid level in serum was significantly negatively correlated with the abundance of *F. nucleatum* (*P* = 0.0026, R = -0.2807), as shown in Fig. 6A. Furthermore, to exclude the possible impact of patients’ renal function, we studied the relationship between the uric acid-to-creatinine ratio and the abundance of *F. nucleatum*. Similarly, a significant negative correlation was observed between the uric acid-to-creatinine ratio and the *F. nucleatum* abundance (*P* = 0.0412, R = − 0.1924), as shown in Fig. 6B. These results support the hypothesis that *F. nucleatum* can lead to a decreased level of uric acid in serum, which is strongly associated with unfavorable survival outcomes in patients.

## Discussion

Metabolic reprogramming is vital for cancer cells with rapid proliferation to meet the energy demand for survival and to adapt to the surrounding microenvironment, which is a hallmark of cancer [14]. Emerging evidence has revealed that *F. nucleatum* can induce metabolic shifts in cancer cells, such as increases in glucose metabolism and glutamine metabolism, which are closely related to tumor occurrence and development [15, 16]. In this study, we showed an altered metabolic profile in HNSCC cells induced by *F. nucleatum* using LS-MS. This alteration was enhanced over time after coculture. The results of KEGG pathway enrichment analysis showed that these significantly changed metabolites were related to several critical metabolic pathways, with purine metabolism as the most significantly enriched pathway. These results supported our previous assumption that the involvement of *F. nucleatum* is vital to the mechanism underlying metabolic reprogramming and poor clinical outcomes in patients with HNSCC.

Uric acid is the end product of purine degradation and is a well-known potent antioxidant in bodily fluids [17]. Although there is still controversy regarding the antitumor effect of uric acid in different types of cancer, our previous study in 814 HNSCC patients revealed that uric acid could serve as an independent prognostic factor for HNSCC [18]. Higher uric acid levels in serum before surgery were significantly correlated with favorable survival outcomes in patients [18]. In line with our previous findings, large prospective population-based studies have shown that a high uric acid level is inversely associated with the risk of cancer and, furthermore, that a high uric acid level can predict better prognosis in patients with cancer [19, 20]. The present study revealed one possible explanation for this phenomenon. *F. nucleatum* could lead to a decreasing trend in purine degradation and thus a decreased uric acid level in HNSCC, which then facilitates tumor progression and results in poor patient outcomes.

Downregulated purine degradation means that more purine nucleotides are available to support cell proliferation. Purine metabolism plays a paramount role in providing substrates for DNA and RNA biosynthesis. Additionally, it generates essential energy materials and cofactors to support cell survival and proliferation [21]. Previous studies have noted aberrant purine metabolism in tumor cells, and antitumor drugs targeting purine metabolism were among the first drugs to be widely used in clinical practice [22, 23]. One feature of this aberrant metabolism is enhanced purine biosynthesis to provide fundamental purine nucleotides to support the uncontrolled proliferation characterizing tumor cells [24]. In our study, *F. nucleatum* induced increases in the AMP and GMP levels in AMC-HN-8 cells, indicating that *F. nucleatum* increases purine supplementation to support cancer proliferation.

Downregulation of the purine degradation pathway is closely related to the altered level of reactive oxygen species (ROS) in cells, which may disrupt the balance between the amounts of ROS generated and scavenged. ROS play a dual role as both tumor suppressors and tumor promoters [25]. From one aspect, ROS can exert their tumor-suppressive effects and serve as a general antitumor mechanism in all treatment modalities except surgery [25, 26]. During purine degradation, hypoxanthine is first oxidized into xanthine, which is the main substrate that is further oxidized into uric acid. It has been verified by in vitro studies that ROS generated via this xanthine oxidase system can exert inhibitory effects on proliferation [27]. From another aspect, as ROS may lead to DNA, RNA, protein and lipid damage, they promote tumor generation and progression [28]. Uric acid is a well-known potent antioxidant that can react with ROS and prevent this damage. Thus, a decreased uric acid level indicates an increased chance for cancer cells to be exposed to ROS, which confers tumor growth advantages. According to the results of our study, the significant increase in the intracellular ROS level triggered by *F. nucleatum* was reversed by uric acid, which may indicate the mechanism underlying the protective role of uric acid in HNSCC.

Our study also indicated that *F. nucleatum* upregulated the de novo biosynthetic pathway in HNSCC. There are two main sources in purine metabolism from which mammalian cells derive purine nucleotides, namely, the complementary salvage pathway and the de novo biosynthetic pathway, and a balance is maintained between these pathways in cells [29]. As described in previous studies, when there is an emerging requirement for purine nucleotides, as occurs in tumor cells, the de novo biosynthetic pathway is the basis for replenishing the purine pool [30]. Elevated levels of enzymes involved in the de novo biosynthetic pathway, such as PPAT and PAICS, have been observed in lung cancer and may serve as potential prognostic markers [31]. Although metabolites related to the de novo biosynthetic pathway were not detected in our study, we discovered that the levels of three critical metabolites in the complementary salvage pathway—hypoxanthine, adenosine and adenine—were significantly decreased, indicating that increased AMP synthesis may originate via the de novo biosynthetic pathway.

## Conclusion

Overall, our study has significant implications for understanding the mechanism by which *F. nucleatum* alters the metabolic profile in HNSCC. Most importantly, *F. nucleatum* triggers obviously aberrant purine metabolism and an imbalance in intracellular ROS, leading to tumor progression and poor prognosis in patients, which can be reversed by uric acid. These findings indicate the possibility of targeting *F. nucleatum*-induced purine metabolism reprogramming in the future treatment of HNSCC.

## Data Availability

Data availability on request from the author
